# Characterization of Latex-Clearing Protein and Aldehyde Dehydrogenases Involved in the Utilization of poly(*cis*-1,4-isoprene) by *Nocardia farcinica* NBRC 15532

**DOI:** 10.3390/microorganisms10122324

**Published:** 2022-11-24

**Authors:** Natsuhei Suzuki, Daito Suda, Nguyen Thi Thuy Ngan, Namiko Gibu, Nguyen Lan Huong, To Kim Anh, Daisuke Kasai

**Affiliations:** 1 Department of Materials Science and Bioengineering, Nagaoka University of Technology, Nagaoka 940-2188, Japan; 2 School of Biotechnology and Food Technology, Hanoi University of Science and Technology, Hanoi 10000, Vietnam

**Keywords:** poly(*cis*-1,4-isoprene) utilization, *Nocardia farcinica*, latex-clearing protein, aldehyde dehydrogenase

## Abstract

Microbial degradation of natural rubber and synthetic poly(*cis*-1,4-isoprene) is expected to become an alternative treatment system for waste from poly(*cis*-1,4-isoprene) products including scrap tires. *Nocardia farcinica* NBRC 15,532, a gram-positive rubber-degrading bacterium, can utilize poly(*cis*-1,4-isoprene) as the sole source of carbon and energy to produce oligo-isoprene metabolites containing aldehyde and keto end groups. A homology-based search of the genome revealed a gene encoding a latex-clearing protein (Lcp). Gene disruption analysis indicated that this gene is essential for the utilization of poly(*cis*-1,4-isoprene) in this strain. Further analysis of the genome sequence identified aldehyde dehydrogenase (ALDH) genes as potential candidates for oxidative degradation of oligo-isoprene aldehydes. Based on the enzymatic activity of the ALDH candidates, NF2_RS14000 and NF2_RS14385 may be involved in the degradation of oligo-isoprene aldehydes. Analysis of the reaction products revealed that these ALDHs oxidized tri- to penta-isoprene aldehydes, which were generated by the reaction of Lcp. Based on the inability of ALDH gene deletion mutants, we concluded that NF2_RS14000 is mainly involved in the utilization of poly(*cis*-1,4-isoprene) and the oxidative degradation of oligo-isoprene aldehydes in *Nocardia farcinica* NBRC 15,532.

## 1. Introduction

Natural rubber (NR) derived from *Hevea brasiliensis* [1] primarily consists of poly(*cis*-1,4-isoprene). NR and synthetic polyisoprene rubber (IR) are important raw materials for industrial products such as automotive tires and medical gloves. However, waste from poly(*cis*-1,4-isoprene) products is difficult to recycle and typically treated in landfills or through combustion processes [2]. The biotransformation method is anticipated to be an alternative eco-friendly treatment process for poly(*cis*-1,4-isoprene) containing waste, and research on the establishment of a poly(*cis*-1,4-isoprene) conversion system utilizing microorganisms is currently being conducted to build a sustainable, low-carbon society [3,4].

**Figure 1:**
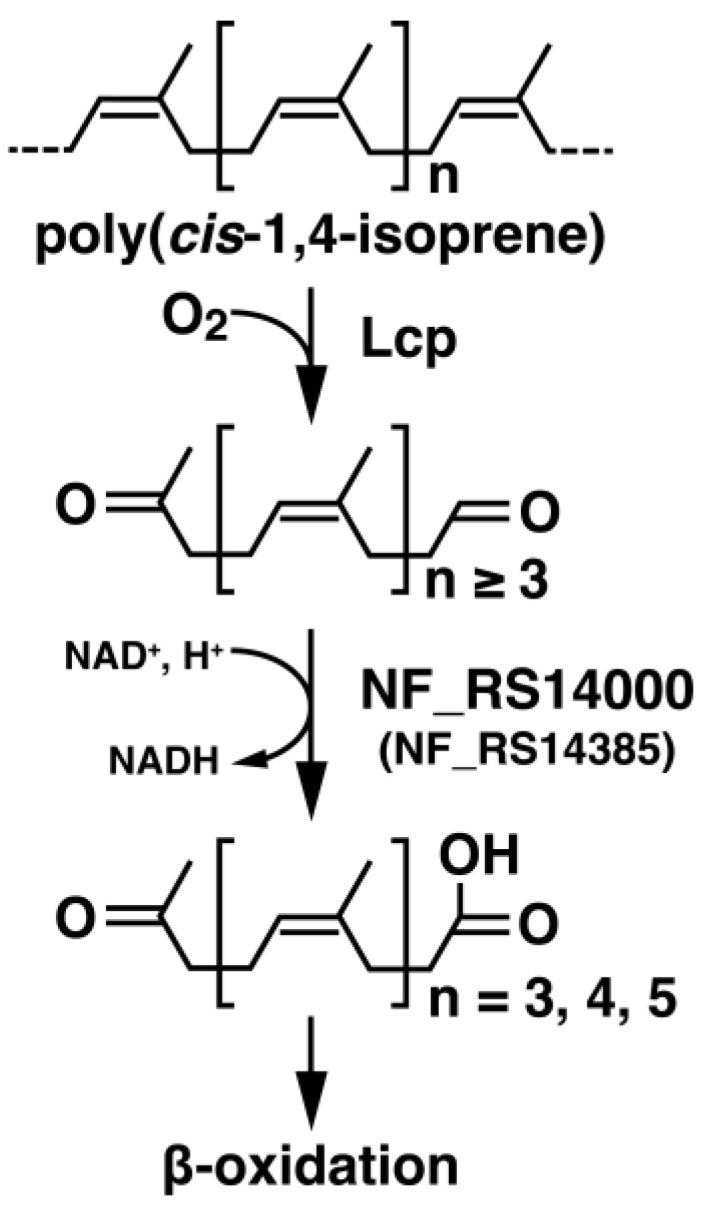
Proposed catabolic pathway for poly(*cis*-1,4-isoprene). Poly(*cis*-1,4-isoprene) is cleaved to form oligo-isoprenoids with aldehyde and keto end groups (oligo-isoprene aldehydes). The tri- to penta-isoprene aldehydes are oxidized to oligo-isoprene acids by NF2_RS14000 and NF2_RS14385 in strain NBRC 15532.

The biodegradation of poly(*cis*-1,4-isoprene) has been shown to begin with the depolymerization of the poly(*cis*-1,4-isoprene) into low-molecular-weight products (oligo-isoprene aldehydes) with aldehyde and keto end groups by the addition of oxygen by extracellular oxygenases (Figure 1) [5,6,7,8,9,10,11,12,13]. A latex-clearing protein (Lcp), a poly(*cis*-1,4-isoprene)-degrading oxygenase, has been identified in NR-degrading gram-positive bacteria (actinomycetes) [6,14,15,16]. In contrast, gram-negative bacteria, such as *Rhizobacter gummiphilus* NS21^T^ and *Steroidobacter cummioxidans* 35Y have other types of rubber oxygenases (RoxA and RoxB) [11,17,18,19]. The aldehyde group of the resulting low-molecular-weight isoprenoids is thought to be oxidized by the putative heterodimeric molybdenum hydroxylase, OxiAB before entering the β-oxidation pathway in *Streptomyces* sp. strain K30 [20]. A putative twin-arginine translocation signal sequence was identified in the amino-terminal region of OxiB, suggesting that OxiAB is an extracellular enzyme. In *Gordonia polyisoprenivorans* VH2, which does not have the OxiAB coding genes, two aldehyde dehydrogenases (ALDHs), GPOL_c02580 (AFA71331.1) and GPOL_c37100 (AFA74722.1), were found [21]. GPOL_c02580 and GPOL_c37100 are capable of oxidizing oligo-isoprene aldehydes in this strain. However, the genes and their gene products responsible for the oxidation of oligo-isoprene aldehydes in other rubber degraders such as *Rhodococcus*, *Actinoplanes*, *Nocardia*, and gram-negative bacteria, have not yet been characterized. Moreover, the reaction products of oligo-isoprene aldehydes obtained by the oxidation of OxiAB and ALDHs have not been identified to date. To gain insights into the entire biodegradation pathway of poly(*cis*-1,4-isoprene), it is important to identify the degradation products of oligo-isoprene aldehydes.

In *Nocardia*, a *lcp* homologous gene that is responsible for the poly(*cis*-1,4-isoprene) degradation has been identified [14,22,23]. However, the gene for the degradation of oligo-isoprene aldehydes has not been identified. Here, *Nocardia farcinica* NBRC 15,532 which is able to grow on NR and IR as a sole source of carbon and energy, was obtained from a stock culture at the Biological Resource Center, National Institute of Technology and Evaluation (NITE; Tokyo, Japan). Based on the genome sequence analysis of strain NBRC 15532, an *lcp* homologous gene and the aldehyde dehydrogenase genes that are responsible for NR and IR utilization in this strain were identified. The results uncovered the role of the poly(*cis*-1,4-isoprene) degradation pathway genes, including *lcp* and aldehyde dehydrogenase genes at the molecular level, in strain NBRC 15532.

## 2. Materials and Methods

### 2.1. Bacterial Strains and Culture Conditions

*N. farcinica* NBRC 15,532 and its mutant derivatives were routinely grown at 37 °C in PYM medium (0.5% bacto peptone, 0.3% yeast extract, and 0.1% MgSO_4_ 7H_2_O; pH 7.0), LBP medium (2% bacto peptone, 1% yeast extract, 2% NaCl), or W minimal salt medium [24] containing 10 mM sodium succinate or 1% IR. *Escherichia coli* strains were cultivated at 37 °C in LB medium (2% bacto tryptone, 1% yeast extract, 1% NaCl). If necessary, the medium was supplemented with 100 mg/L ampicillin (AMP), 25 mg/L kanamycin (KAN), 25 mg/L nalidixic acid (NAL), and 50 mg/L neomycin (NEO).

### 2.2. DNA Manipulation, Nucleotide Sequencing, and Sequence Analysis

DNA manipulations, including total DNA isolation and nucleotide sequencing, were performed as previously described [25]. Nucleotide sequence analysis was performed using MacVector software (MacVector, Inc., Cary, NC), as previously described [26]. The genome sequence of NBRC 15,532 was used to identify poly(*cis*-1,4-isoprene) utilization genes in the NBRC 15,532 genome database (https://www.ncbi.nlm.nih.gov/nuccore/NZ_BDBJ00000000.1, accessed on 15 March 2021). Signal sequences were predicted using SignalP 6.0 software (https://services.healthtech.dtu.dk/service.php?SignalP, accessed on 14 November 2022) [27].

### 2.3. Expression of His-Tagged Lcp and Aldehyde Dehydrogenase Genes in E. coli

The coding regions of the Lcp and aldehyde dehydrogenase genes were amplified by PCR using the primers listed in Table 1. Each PCR-amplified fragment was cloned into the expression vector pColdI using in-fusion cloning. The resultant plasmids were independently introduced into *E. coli* BL21(DE3) and the transformed cells were grown in 100 mL of LB medium containing AMP at 30 °C. When the absorbance at 600 nm (*A*_600_) of the culture reached 0.5, it was incubated at 15 °C for 30 min and cultivated again at 15 °C for 24 h after the addition of 0.1 mM isopropyl-β-D-thiogalactopyranoside. After the incubation, the crude extracts were prepared by using an ultrasonic disrupter, as described previously [28]. His-tagged proteins were purified using a HiTrap TALON superflow column (Cytiva, Uppsala, Sweden), according to a previous method [16].

### 2.4. Enzyme Assays

#### 2.4.1. Lcp

The substrate-dependent oxygen consumption rate was measured to determine the activity of purified Lcp. A 4-mL assay mixture contained 50 mM phosphate buffer (pH 7.4), NR latex (final concentration 0.5%), and purified Lcp (20 μg of protein). The reaction mixture was incubated at 35 °C and the oxygen consumption rate was determined using an oxygen electrode (FireSting O2-C; BAS Inc., Tokyo, Japan). One unit of enzyme activity was defined as the amount of activity that resulted in the consumption of 1 μmol of O_2_/min. Specific activity is expressed as units per milligram of protein.

#### 2.4.2. Oligo-Isoprene Aldehyde Dehydrogenase

Oligo-isoprene aldehyde dehydrogenase was assayed in a similar way as previously reported [21]. To obtain oligo-isoprene aldehydes, 500 μL of the reaction mixture containing 0.8% (*v*/*v*) PSS-pio800 (Polymer Standards Service GmbH, Mainz, Germany) and 20 μg of purified Lcp protein was incubated at 35 °C for 12 h. After the reaction, 500 μL of 50 mM Tris-HCl (pH 7.0) containing 50 µg protein of each purified ALDH and 200 µM NAD^+^ was added to the mixture, which was then incubated at 30 °C. After 1 h, 120 µM 2,6-dichlorophenolindophenol (DCPIP) and 20 µM phenazine methosulfate (PMS) were added. Enzyme activity was determined spectrophotometrically by monitoring the decrease in absorbance at 660 nm, derived from the consumption of DCPIP. Specific activity was calculated as the concentration of the remaining DCPIP using molar extinction coefficients of 20,460 M^−1^ cm^−1^ for DCPIP.

### 2.5. Determination of Oligo-Isoprene Aldehydes and Acids

To determine oligo-isoprene aldehydes and acids, an enzymatic reaction mixture was extracted with pentane, dried in vacuo, and dissolved in 2 mL of methanol. Then, 5 μL of the extract was subjected to a liquid chromatography-mass spectrometry (LC–MS) system (Infinity Lab LC/MSD; Agilent Technology Inc., Santa Clara, CA, USA) equipped with a ZORBAX SB-C18 2.1 × 50 mm column (Agilent Technology). LC–MS analysis was. performed as described previously [16].

### 2.6. Construction of Deletion Mutants

Each *lcp* and aldehyde dehydrogenase gene was deleted using the *sacB* counterselection system as described previously [29,30,31]. The oligonucleotides that amplified the flanking regions of each gene are listed in Table 1. The amplified fragments were connected and inserted into the pK18*mobsacB* [30]. Each resulting plasmid was introduced into NBRC 15532, and transformants were selected using NEO resistance and sucrose sensitivity as described previously [16]. To obtain a deletion mutant using the *sacB* counterselection system, the sucrose-sensitive and NEO-resistant transformants were subjected to a second selection on a sucrose-containing 0.2 × LB agar plate. Deletion of the genes was confirmed by diagnostic PCR using specific primer sets, and subsequently by DNA sequencing of the PCR-amplified regions flanking the deletion.

### 2.7. Quantitative Reverse Transcription-PCR (qRT-PCR) Analysis

NBRC 15,532 cells were grown in W medium containing 10 mM sodium succinate with or without 1% IR at 37 °C for 5 d. Total RNA was extracted from the resulting cells using ISOGEN II (Nippon Gene Co., Ltd., Tokyo, Japan), according to the manufacturer’s instructions. Single-stranded cDNA was synthesized from 1 µg of total RNA after treatment with RNase-free DNase I (Roche) as described previously [15]. qRT-PCR analysis was carried out using 50 ng of a cDNA, 4 pmol of specific primer pairs (Table 1), and 10 μL of Fast SYBR Green Master Mix (Life Technologies) in a total reaction volume of 20 μL, according to the previous method [11]. To normalize the quantity of RNA in each sample, the 16S rRNA gene was used as an internal standard.

## 3. Results and Discussion

### 3.1. Characterization of Lcp-Coding Gene of Strain NBRC 15532

A tBLASTn homology search of the genome sequence of NBRC 15,532 was performed using the amino acid sequence of Lcp (API85527) of Nocardia sp. strain NVL3 [14] as the query, and an *lcp* gene (NF2_RS04895) was identified. The deduced amino acid sequence of the lcp gene had an overall identity of 78% and 56% with Lcps from strains NVL3 and K30 (AAR25849), respectively. To determine whether the gene is involved in poly(cis-1,4-isoprene) degradation, 10× histidine-tag-fused (His-tagged) lcp was expressed in E. coli BL21(DE3). SDS-polyacrylamide gel electrophoresis (SDS-PAGE) analysis revealed the production of a 46-kDa protein (Figure S1), which is consistent with the deduced amino acid sequence. When purified His-tagged Lcp was incubated with NR latex, oxygen consumption activity was observed at a specific activity of 0.50 ± 0.07 U/mg of protein (35 °C, pH 7.5). No consumption of oxygen was observed without protein or NR latex, indicating that the enzyme was required for poly(cis-1,4-isoprene) degradation. The optimal temperature and pH for oxygen consumption activity of this enzyme with NR latex were 35 °C and 7.5, respectively. The activity of Lcp from NBRC15532 is slightly lower than those of other reported Lcps in K30 (4.6 U/mg), Actinoplanes sp. OR16 (4.0 U/mg), G. polyisoprenivorans VH2 (1.3 U/mg), and Rhodococcus rhodochrous RPK1 (3.1 U/mg) [10,15,32,33]. Furthermore, although NBRC15532 has sole gene encoding Lcp, it exhibits the same level of NR degradation as other known NR-degrading bacteria. It might be due to the expression level of *lcp* in each NR-degrading bacterium. However, the transcription level of the lcp gene have not been compared, a detailed analysis for the transcription of *lcp* is necessary to clarify the relationship between the degradation activity of NR degrader and the enzymatic activity of Lcp in the future.

To determine the degradation product of poly(cis-1,4-isoprene) by the reaction of Lcp, the reaction mixture containing Lcp and IR was incubated at 35 °C for 12 h and then analyzed by HPLC-ESI-MS. As shown in Figure S2, the appearance of the peak for the protonated molecular ion [M+H]^+^ of oligo-isoprene aldehydes corresponding to molecular sizes from C_20_ to C_50_ was observed. Multiple degradation products of different molecular sizes were produced, suggesting that Lcp, like Lcp in other actinomycetes, randomly cleaves poly(cis-1,4-isoprene) into a mixture of tri-isoprene aldehyde (C_20_) or higher with aldehyde and keto functional groups at the ends [10,32,34].

**Figure 2:**
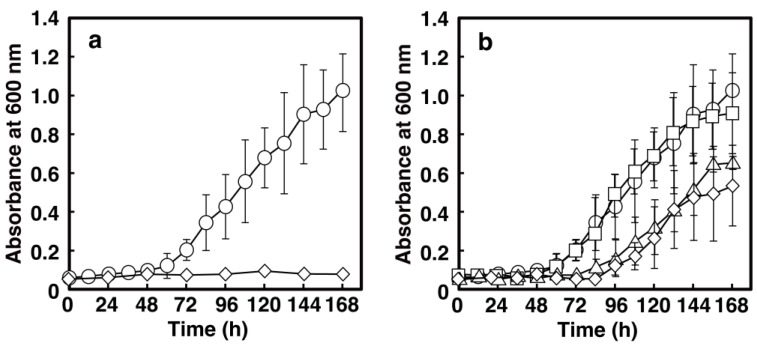
Growth of NBRC 15,532 and its deletion mutants on IR. Cells were grown in W medium containing 1.0% IR. NBRC 15,532 (circles) and lcp deletion mutant (diamonds) were shown in (**a**). NBRC 15,532 (circles), Δ14000 (triangles), Δ14385 (squares) and Δ14000-Δ14385 double mutant (diamonds) were shown in (**b**). The data are averages ± standard deviations of three independent experiments performed in parallel.

To examine the role of the lcp gene in poly(cis-1,4-isoprene) utilization by NBRC 15532, the gene was inactivated by an internal deletion using a gene replacement technique. As shown in Figure 2a, the deletion mutant strain did not grow on IR. When the cells of NBRC 15,532 were grown with the pieces of the DPNR glove, bacterial colonies and pronounced pitting on the glove were observed on the surface of the glove pieces after 15 d of incubation (Figure S3). By contrast, no colonies or pits were found in the case of the lcp deletion mutant. These results indicated that the lcp gene is essential for the utilization of poly(cis-1,4-isoprene) in NBRC 15532.

### 3.2. Identification of ALDH for the Oxidation of Oligo-Isoprene Aldehydes

Oligo-isoprene aldehyde dehydrogenases have been reported only in *G. polyisoprenivorans* strain VH2 [21]. When a homology search of the genome sequence of strain NBRC 15,532 was performed using the amino acid sequence of GPOL_c02580 from strain VH2 as a query, seven putative ALDH genes were predicted. ALDH activity toward oligo-isoprene aldehydes in strain NBRC 15,532 was 0.12 mU/mg when NAD^+^ was used as coenzyme. However, the activity when NADP^+^ was used as a coenzyme was approximately 10% of that with NAD^+^. Based on these results, we considered that NAD^+^ is mainly used as a cofactor for oligo-isoprene aldehyde oxidation in the strain NBRC 15,532 and compared the enzymatic activity of seven ALDH candidates when NAD^+^ was used as a cofactor.

**Figure 3:**
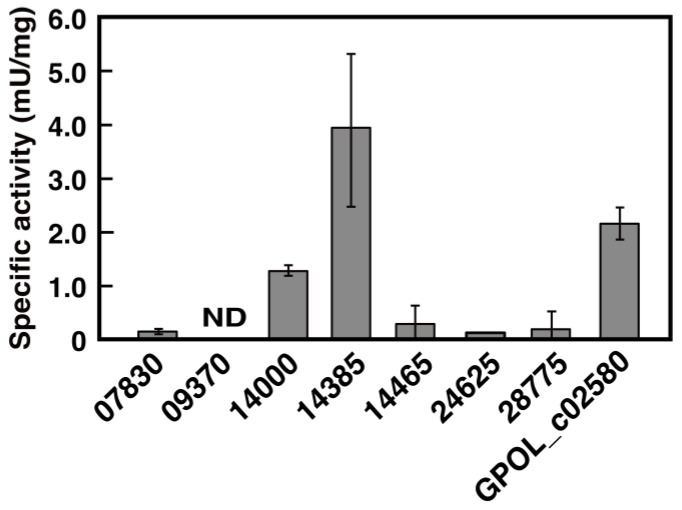
ALDH activities of ALDH candidates toward oligo-isoprene aldehydes. The assay was performed basically as described previously [21]: 500 μL of 50 mM Tris-HCl (pH 7.0) containing 50 µg protein of each purified ALDH and 200 µM NAD^+^ were added to 500 μL of the Lcp reaction mixture containing IR. After 1 h of reaction, absorbance at 660 nm derived from DCPIP was measured to evaluate the enzymatic activity. The data are the mean values ± standard deviations of four independent experiments. ND; not detected.

To examine the activity of ALDHs toward oligo-isoprene aldehydes, each ALDH gene was expressed as a His-tagged protein in *E. coli* BL21(DE3). Using SDS-PAGE analysis, each protein was specifically observed in the crude extracts of *E. coli* BL21(DE3) harboring each expression plasmid, and their sizes were consistent with the size estimated from each deduced amino acid sequence of the ALDH genes (Figure S4). To characterize enzymatic activity, each His-tagged protein was purified by Ni-affinity column chromatography. Oligo-isoprene aldehydes were prepared as substrates by reacting poly(*cis*-1,4-isoprene) with purified Lcp for 12 h. After the Lcp reaction, purified ALDH and 200 μM NAD^+^ were added to the reaction mixture to react with the oligo-isoprene aldehydes. As shown in Figure 3, significant degradation activities were observed for two gene products, NF2_RS14000 and NF2_RS14385. The specific activities of NF2_RS14000 and NF2_RS14385 were 1.2 and 3.9 mU/mg, respectively. These specific activities were comparable to that of GPOL_c02580 (2.1 mU/mg) of strain VH2. Other ALDH candidates, excluding NF2_RS09370, exhibited weak activity in the presence of oligo-isoprene aldehydes. Furthermore, NF2_RS09370 showed no activity under the same conditions, suggesting that NF2_RS14000 and NF2_RS14385 are important for the degradation of oligo-isoprene aldehydes in NBRC 15532.

The deduced amino acid sequences of NF2_RS14000 and NF2_RS14385 showed 34–40% identity with those of GPOL_c02580, GPOL_c37100, and geranial dehydrogenase (H1ZV37) of *Castellaniella defragrans* [35]. The deduced amino acid sequence of NF2_RS14000 exhibited 73% identity with that of Ald1 (Q9FDS1) from *Acinetobacter* sp. strain M-1, which is involved in the oxidation of tetradecanal [36]. NF2_RS14385 shares a relatively high identity (43%) with retinal dehydrogenase (NP_033048.2) from *Mus musculus* [37,38], which oxidizes retinal, including the isoprene-unit and terminal aldehyde groups. Based on sequence similarities, NF2_RS14385 and NF2_RS14000 may be involved in the oxidation of carbon chains with terminal aldehyde groups to fatty acids. As no signal peptide sequence was found in the N-terminal amino acid sequence regions of NF2_RS14000 and NF2_RS14385, these gene products appear to be intracellular enzymes.

### 3.3. Transcriptional Induction of the lcp and the ALDH Genes

**Figure 4:**
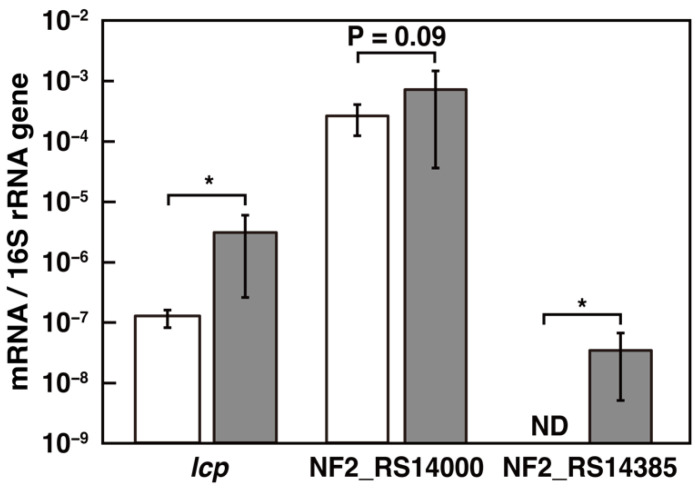
Quantification of the expression levels of the *lcp*, NF2_RS14000, and NF2_RS14385 genes. Total RNA was isolated from NBRC 15,532 cells grown in W medium containing 10 mM succinate with IR (gray bars) or without IR (open bars). mRNA expression levels were calculated as the ratio against 16S rRNA gene expression. The data are the mean values ± standard deviations of five independent experiments. Statistical analysis was performed using Student’s *t* test. The asterisks indicate statistically significant differences between the values linked by brackets (* *p* < 0.05). ND; not detected.

To determine whether transcription of the *lcp*, NF2_RS14000, and NF2_RS14385 genes was induced during the utilization of poly(*cis*-1,4-isoprene), the mRNA levels of these genes were measured by qRT-PCR analysis. Total RNA was harvested from the cells of NBRC 15,532 grown on succinate with or without 1% IR. The transcriptional level of *lcp* in cells grown with IR was 21-fold higher than in cells grown without IR (*p* < 0.05, Student’s *t* test) (Figure 4). It has been suggested that lcp transcription is induced during poly(*cis*-1,4-isoprene) utilization. Furthermore, the transcription of NF2_RS14385 was induced during the growth of NBRC 15,532 with IR (Figure 4). By contrast, the NF2_RS14000 gene is constitutively transcribed in NBRC 15,532 cells. However, the transcriptional level of NF2_RS14000 was shown to be more than 1000-fold higher during growth in the presence of IR than NF2_RS14385. Since NF2_RS14000 seems to be significantly more abundant in cells in the presence of IR than NF2_RS14385, NF2_RS14000 is thought to be mainly responsible for poly(*cis*-1,4-isoprene) utilization. According to the constitutive expression of NF2_RS14000, the aldehyde compounds generated during poly(*cis*-1,4-isoprene) utilization appear to be rapidly oxidized to fatty acids. Because many types of aldehyde compounds have been found to have cytotoxic potential [39,40,41], the rapid degradation of aldehyde compounds is thought to be important for the utilization of poly(*cis*-1,4-isoprene).

### 3.4. Disruption of the ALDH Genes in NBRC 15532

To clarify the involvement of NF2_RS14000 and NF2_RS14385 in poly(*cis*-1,4-isoprene) utilization, each ALDH gene was disrupted by gene replacement using homologous recombination. To compare the growth rates of NBRC 15,532 and each ALDH gene deletion mutant on IR, each strain was incubated on W medium with IR as a carbon source. Comparison of the growth of each deletion mutant with that of the wild-type strain showed that the growth rate of the NF2_RS14000 deletion mutant (Δ14000) was significantly decreased (Figure 2b). By contrast, growth of the NF2_RS14385 deletion mutant (Δ14385) was slightly decreased. In addition, the growth of the double-deletion mutant was further decreased compared to that of Δ14000. However, it did not completely lose its growth ability, suggesting that NF2_RS14000 and NF2_RS14385 are indeed involved in IR utilization, but are not essential for the growth of NBRC 15,532 on IR.

**Figure 5:**
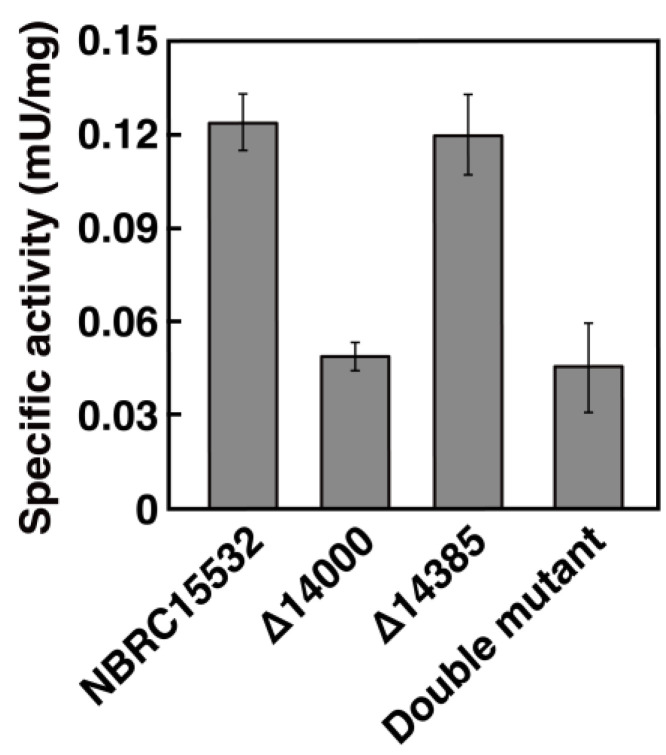
ALDH activities of NBRC 15,532 and its deletion mutants toward oligo-isoprene aldehydes. The reaction mixture (1 mL) containing 1 mg protein of the crude extract and 200 µM NAD^+^ were incubated for 1 h. After the reaction, absorbance at 660 nm derived from DCPIP was measured to evaluate the ALDH activity. The data are the mean values ± standard deviations of four independent experiments.

To estimate the level of participation of each ALDH gene in oligo-isoprene aldehyde degradation, the ALDH activities of Δ14000 and Δ14385 cell extracts grown on IR were determined. When the cell extract of Δ14000 was reacted with oligo-isoprene aldehydes prepared from poly(*cis*-1,4-isoprene) in the presence of NAD^+^, the activity of Δ14000 was approximately 40% that of the wild-type strain (Figure 5). By contrast, the activity of Δ14385 was comparable to that of the wild type. In addition, the activity of the double-deletion mutant was almost the same as that of Δ14000, suggesting that NF2_RS14000 was mainly involved in the oxidation of oligo-isoprene aldehydes in NBRC15532. The double-deletion mutant did not completely lose its ability to grow on IR and degrade oligo-isoprene aldehydes, raising the possibility that unidentified enzymes are involved in the oxidation of oligo-isoprene aldehydes in strain NBRC 15532. In this study, ALDH activity toward oligo-isoprene aldehydes remained despite the disruption of NF2_RS14000 and NF2_RS14385, which had significant ALDH activity toward oligo-isoprene aldehydes. This means that other ALDHs, whose exact oxidation activity toward oligo-isoprene aldehydes was not detected in heterologous host expression, might act in the cells of NBRC 15532. It is necessary to express ALDH candidates using hosts closely related to the genus *Nocardia* and examine ALDH activity toward oligo-isoprene aldehydes in the future.

### 3.5. Identification of the Reaction Product of Oligo-Isoprene Aldehydes

**Figure 6:**
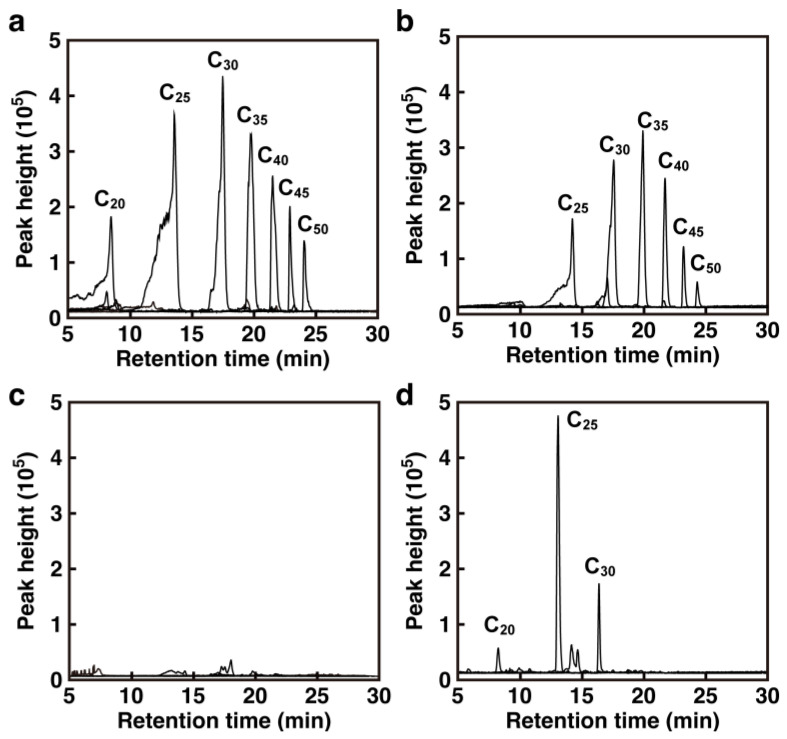
Extracted ion chromatogram of the degradation products of oligo-isoprene aldehydes. The reaction mixture containing oligo-isoprene aldehydes produced by the Lcp reaction, 150 μg of purified NF2_RS14000 and 200 μM NAD^+^ were incubated: (**a**,**c**) and (**b**,**d**) showed extracted ion chromatogram of the reaction products at the start of incubation and after 12-h incubation, respectively. Peaks for extracted ions of the oligo-isoprene aldehydes and acids were shown in (**a**,**b**) and (**c**,**d**), respectively.

Because the analysis of gene deletion mutants revealed that NF2_RS14000 is mainly involved in the utilization of poly(*cis*-1,4-isoprene), the reaction products of oligo-isoprene aldehydes by the NF2_RS14000 gene product were examined. To identify the reaction products, purified NF2_RS14000 gene product was added to the reaction mixture containing oligo-isoprene aldehydes produced by Lcp (Figure 6). After 12 h of reaction, the intensities of the peaks at m/z 305.2 and 373.3, corresponding to [M+H]^+^ of tri- (C_20_) and tetra- (C_25_) oligo-isoprene aldehydes, respectively, were significantly decreased (Figure 6b). In this reaction mixture, the generation of peaks of *m/z* 321.2, 389.3, and 457.3 corresponding to [M+H]^+^ of tri- (C_20_), tetra- (C_25_), and penta- (C_30_) oligo-isoprene acids, respectively, was observed (Figure 6d). The tri- (C_20_) to penta- (C_30_) oligo-isoprene aldehydes were oxidized to the corresponding oligo-isoprene acids by NF2_RS14000. Similar conversion profile was found in the case of NF2_RS14385, suggesting that poly(*cis*-1,4-isoprene) is utilized via tri- (C_20_) to penta- (C_30_) oligo-isoprene acids before entering the β-oxidation pathway in strain NBRC 15532.

## 4. Conclusions

In this study, the gene code for Lcp, which is directly involved in poly(*cis*-1,4-isoprene) utilization, was identified. Based on the analysis of the reaction products, poly(*cis*-1,4-isoprene) was degraded to C_20_–C_50_ oligo-isoprene aldehydes by the Lcp reaction. NF2_RS14000 and NF2_RS14385 were identified as ALDH for the oxidation of oligo-isoprene aldehydes generated from poly(*cis*-1,4-isoprene). The generation of C_20_ to C_30_ oligo-isoprene acids as degradation products of oligo-isoprene aldehydes by the NF2_RS14000 and NF2_RS14385 reactions was indicated. Analysis of the gene deletion mutants revealed that NF2_RS14000 was mainly involved in the utilization of poly(*cis*-1,4-isoprene) in NBRC 15532. The ALDHs predicted in this study had no signal peptide sequence. Therefore, we conclude that oligo-isoprene aldehydes produced by the reaction of Lcp outside the cells are oxidized intracellularly by ALDH after uptake into the cell. However, the oligo-isoprene aldehyde degradation and poly(*cis*-1,4-isoprene) utilization abilities of the double-deletion mutant were not completely lost, suggesting that there are other unidentified gene(s) involved in the utilization of poly(*cis*-1,4-isoprene). Therefore, it is necessary to identify the gene(s) to gain a better understanding of poly(*cis*-1,4-isoprene) utilization in this strain.

## Figures and Tables

**Table 1:** Oligonucleotide sequences used in this study *.

Oligo Nucleotide	Sequence (5’ to 3’)
For gene expression	
lcp_Nde_F	GAAGGAGATATACATATGGATGGACTCAGCAGGCG
lcp_Hind_R	GAGTGCGGCCGCAAGCTTGCGATGCGGTTTGGTCA
07830_Nde_F	TCGAAGGTAGGCATATGACCACTTCCGCCCCCACC
07830_Nde_R	GTACCGAGCTCCATATCAGGGTCGGCAGACGTCCT
09370_Nde_F	TCGAAGGTAGGCATATGAACCGATCGATGTCCGTC
09370_Nde_R	GTACCGAGCTCCATATCACACCATGATGTTGATGA
14000_Nde_F	TCGAAGGTAGGCATATGATCTATGCAAAGCCGGG
14000_EcoR_R	CGACAAGCTTGAATTACGGTGATGTGGGTGTGT
14385_Nde_F	TCGAAGGTAGGCATATGACCGACACGCTTTCCGAG
14385_Nde_R	GTACCGAGCTCCATATCACAACTGCGCGTTGATCG
14465_Nde_F	TCGAAGGTAGGCATATGCGAAACCAGCTCTTCATC
14465_Nde_R	GTACCGAGCTCCATATCAGGCCAACGCGGTCCAGA
24625_Nde_F	TCGAAGGTAGGCATAATGCATTACGACAGCTTGTT
24625_EcoR_R	CGACAAGCTTGAATTCTAGCCGGTCCAGCCCAT
28775_Nde_F	TCGAAGGTAGGCATAATGAGCGGACTTCTGCCC
28775_EcoR_R	CGACAAGCTTGAATTTCAGACCGCGGTGGCGAT
02580_VH2_Nde_F	TCGAAGGTAGGCATATGATCACCTACGACAAACTC
02580_VH2_Nde_R	GTACCGAGCTCCATATCAGGCGTAGATCGACTTG
For qRT-PCR	
lcp_F	GATCAGCCAGAACGACATGA
lcp_R	CGAGTTGGGGATGTACTCGT
14000_F	GCACTGATCCACTCCTCCAT
14000_R	CAGGTTCTTGGTCTGCTGGT
14385_F	CGTTCGAGGGTGAATGGTCG
14385_R	TTGCCGTTGTCCAGCGATTC
16S_F	AGAGATGTAGGCCCCCTTGT
16S_R	CCGGTACGGCTACCTTGTTA
For gene disruption	
lcp_UP_F	CGACTCTAGAGGATCGAACACCGAGGAGAGAGAGG
lcp_UP_R	CGACTCTAGAGGATCACGAAGCCGACCAGCTGCGT
lcp_DW_F	CGTGTACTGGCTCTTCGACG
lcp_DW_F	CGGTACCCGGGGATCCGGTGGCGGTGCCCGGCGCT
14000_UP_F	CGGTACCCGGGGATCACCTCGCTTCCGTCGTGG
14000_UP_R	ACCGTAGAGGGTGTCAATGTTGGCGCGCTCGCTCG
14000_DW_F	GACACCCTCTACGGTCTGGG
14000_DW_R	CGACTCTAGAGGATCCCGAGTGGGACACGATCG
14385_UP_F	CGGTACCCGGGGATCGCCCTCGAGCAACTGCTG
14385_UP_R	TAGGGGGTGTCGTTGCACAGCGACCATTCACCCTC
14385_DW_F	CAACGACACCCCCTACGGCC
14385_DW_R	CGACTCTAGAGGATCGGGATGTGGTCCGGATGC

* These primers were constructed in this study.

## Supplementary Materials

The following supporting information can be downloaded at: https://www.mdpi.com/article/10.3390/microorganisms10122324/s1, Figure S1: SDS-PAGE analysis of protein fractions. Proteins were separated on a SDS-12% polyacrylamide gel and stained with Coomassie brilliant blue. Lanes; M, molecular weight markers; 1, crude extract of *E. coli* BL21(DE3) carrying pColdI expression vector; 2, crude extract of *E. coli* BL21(DE3) containing his-tagged *lcp*; 3, purified his-tagged Lcp. Molecular masses are given on the left; Figure S2: Extracted ion chromatogram of oligo-isoprene aldehydes generated from poly(*cis*-1,4-isoprene). After the Lcp reaction, the molecular mass of the reaction products containing oligo-isoprene aldehydes were analyzed by LC–MS The reaction mixture containing 25 mg poly(*cis*-1,4-isoprene) and 150 μg of purified Lcp was incubated for 12 h; Figure S3: The pieces of NR glove after incubated with *N. farcinica* NBRC 15532 (A) and the *lcp* deletion mutant (B). The NR pieces were prepared by cutting NR glove into 1 cm squares. The cells of wild type and its mutant were incubated at 37 °C for 5, 10, and 15 days; Figure S4: SDS-PAGE analysis of protein fractions. Proteins were separated on a SDS-12% polyacrylamide gel and stained with Coomassie brilliant blue. Lanes; M, molecular weight markers; Fractions of crude extract of *E. coli* BL21(DE3) carrying each plasmid vector and purified his-tagged protein are shown in black and red, respectively. Molecular masses are given on the left.
