# Supplementary material for: Characterization of Latex-Clearing Protein and Aldehyde Dehydrogenases Involved in the Utilization of poly(cis-1,4-isoprene) by Nocardia farcinica NBRC 15532
Source: Microorganisms. 2022 Nov 24;10(12):2324. doi: 10.3390/microorganisms10122324 (PMC9782182; doi:10.3390/microorganisms10122324)
Supplement: Supplementary file 1 [file microorganisms-10-02324-s001.zip › microorganisms-2017271-supplementary.pdf]

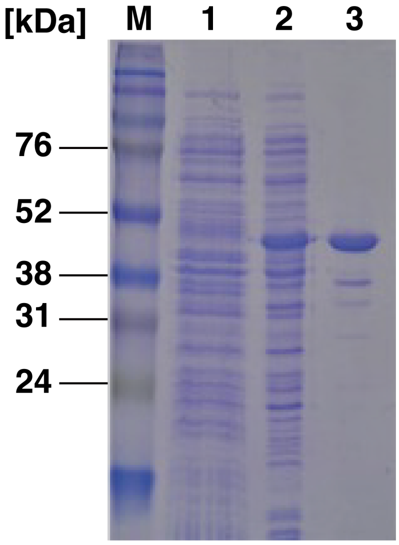

Figure S1. SDS-PAGE analysis of protein fractions. Proteins were separated on a SDS-12% polyacrylamide gel and stained with Coomassie brilliant blue. Lanes; M, molecular weight markers; 1, crude extract of *E. coli* BL21(DE3) carrying pColdI expression vector; 2, crude extract of *E. coli* BL21(DE3) containing his-tagged *lcp*; 3, purified his-tagged Lcp. Molecular masses are given on the left.

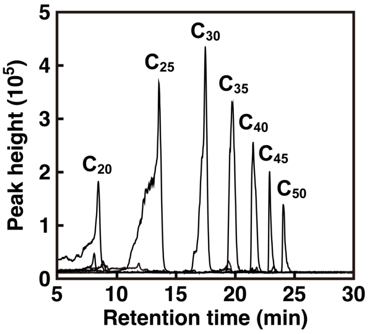

Figure S2. Extracted ion chromatogram of oligo-isoprene aldehydes generated from poly(*cis*-1,4-isoprene). After the Lcp reaction, the molecular mass of the reaction products containing oligo-isoprene aldehydes were analyzed by LC–MS The reaction mixture containing 25 mg poly(*cis*-1,4-isoprene) and 150 μg of purified Lcp was incubated for 12 h.

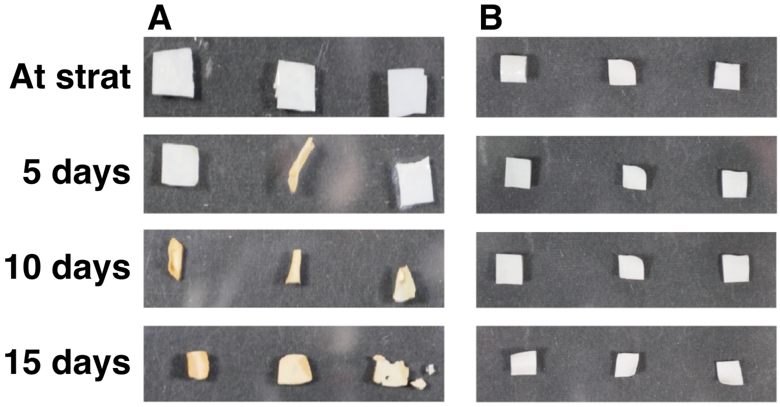

Figure S3. The pieces of NR glove after incubated with *N. farcinica* NBRC 15532 (A) and the *lcp* deletion mutant (B). The NR pieces were prepared by cutting NR glove into 1 cm squares. The cells of wild type and its mutant were incubated at 37°C for 5, 10, and 15 days.

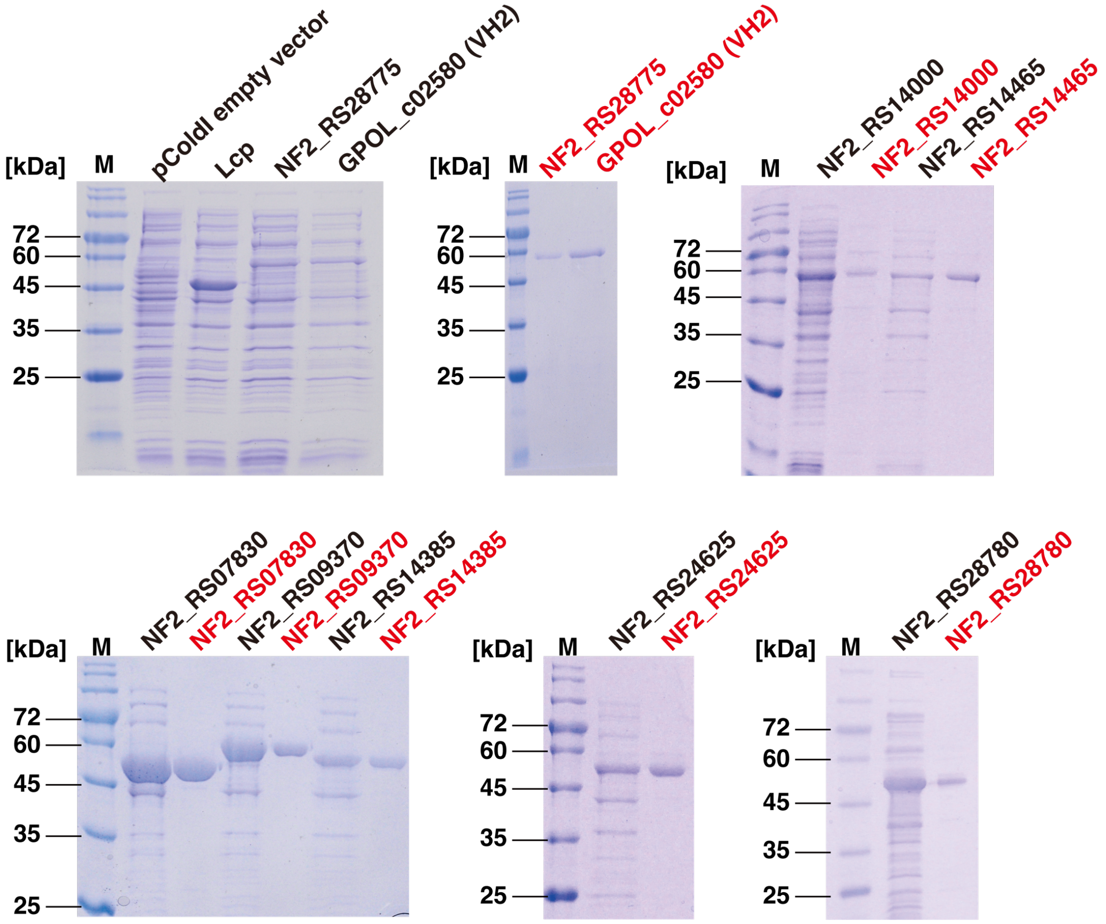

Figure S4. SDS-PAGE analysis of protein fractions. Proteins were separated on a SDS-12% polyacrylamide gel and stained with Coomassie brilliant blue. Lanes; M, molecular weight markers; Fractions of crude extract of *E. coli* BL21(DE3) carrying each plasmid vector and purified his-tagged protein are shown in black and red, respectively. Molecular masses are given on the left.
